# Acute Myeloid Leukemia Secondary to Radiation Therapy for Rectal Cancer: A Case Report

**DOI:** 10.1002/cnr2.70379

**Published:** 2025-12-09

**Authors:** Ahmad Alkhaledi, Laila Alhaj Hussaen, Linda Kahla, Baraa Sammoud, Suha Giselle Ghanem

**Affiliations:** ^1^ Oncology Department Faculty of Medicine, Damascus University Damascus Syria; ^2^ Faculty of Medicine Damascus University Damascus Syria; ^3^ Faculty of Medicine Iuliu Hațieganu University of Medicine and Pharmacy Cluj‐Napoca Romania

**Keywords:** radiation therapy, rectal cancer, t‐AML, therapy‐related acute myeloid leukemia

## Abstract

**Background:**

Therapy‐related AML (t‐AML) is a subtype of acute myeloid leukemia (AML) that arises in the bone marrow and primarily affects white blood cells. It is associated with prior exposure to cytotoxic agents, including chemotherapy and radiotherapy. The risk of the disease increases with age and treatment intensity. Although t‐AML represents 25%–30% of all AML cases, its occurrence following radiotherapy is relatively rare. Diagnosis is confirmed via bone marrow biopsy, with differential diagnoses including other leukemias, lymphomas, and myelodysplastic syndromes. Prognosis is generally poor. Allogeneic hematopoietic stem cell transplantation (allo‐HSCT) remains the most effective curative option, though elderly patients often have limited eligibility due to comorbidities and poor performance status.

**Case:**

Five years after treatment for stage IIA rectal cancer, including radiotherapy, chemotherapy, and surgery, a 77‐year‐old male smoker presented with fatigue, weight loss, progressive dyspnea, headache, dizziness, blurred vision, and melena. Physical examination revealed pallor, purpura, skin crusts, and pustules. Laboratory findings showed anemia, thrombocytopenia, and circulating blasts. Bone marrow biopsy confirmed lymphoid infiltration. He was diagnosed with therapy‐related AML (t‐AML). Despite receiving three cycles of Azacitidine, he died of septic shock 7 months later.

**Conclusion:**

This case highlights the rare development of therapy‐related acute myeloid leukemia in a colorectal cancer survivor following combined pelvic radiation and capecitabine plus oxaliplatin (CAPOX) chemotherapy, underscoring the importance of long‐term hematologic surveillance in such patients.

## 1 Introduction

The World Health Organization (WHO) defines “secondary acute myeloid leukemia” (s‐AML) as acute myeloid leukemia that develops as a late consequence of cytotoxic chemotherapy (CHT) and/or radiation therapy (RT) for either malignant or non‐malignant diseases [1].

S‐AML can be further categorized into two separate categories: AML emerging from antecedent hematological disease (AHD‐AML) and therapy‐associated AML (t‐AML) [2]. S‐AML accounts for 25%–30% of all AML cases and is considered a biologically complex and heterogeneous disease [3, 4].

Myelodysplastic syndromes (MDS) in young patients (aged 18 years or younger) are rare, with an annual incidence of 1–4 cases per million. However, sporadic MDS is predominantly observed in the elderly, with an incidence exceeding 36 per 100 000 in individuals aged 80 years or older [5, 6].

The pathogenesis of secondary AML often involves DNA damage induced by cytotoxic agents such as radiation and chemotherapy, which cause double‐strand breaks and the loss of DNA segments [7].

The time from initial exposure to therapy to the onset of secondary AML can vary significantly, ranging from several months to years. This period is influenced by factors such as the specific cytotoxic agents used, the total dose, and the intensity of prior treatments [8].

Among solid tumors, t‐AML is most frequently reported after treatment for breast, ovarian, and hematologic malignancies. In contrast, according to SEER data, colorectal cancer has not been consistently associated with an increased risk of t‐AML, with studies suggesting little or no excess risk compared to other cancers [9]. Reports of AML following rectal or colon cancer treatment are therefore rare, particularly when both chemotherapy and radiotherapy have been used.

Currently, pathological biopsy remains the gold standard for diagnosing s‐AML [10]. Differential diagnosis for s‐AML includes conditions with overlapping symptoms, such as acute lymphoblastic leukemia, anemia, aplastic anemia, B‐cell lymphoma, chronic myelogenous leukemia, lymphoblastic lymphoma, myelodysplastic syndromes (MDS), myelopathic anemia, and primary myelofibrosis [11].

The prognosis for patients with s‐AML is generally poor, particularly when treated with standard chemotherapy or hematopoietic stem cell transplantation (HSCT). Although the prognosis is unfavorable, HSCT has been shown to be an effective treatment option for younger patients with adverse‐risk cytogenetics [12].

For s‐AML patients, the standard chemotherapy regimen consists of the “7 + 3” protocol, which includes a 7‐day intravenous infusion of cytarabine and a 3‐day infusion of an anthracycline (e.g., daunorubicin or idarubicin). Complete remission rates with this regimen range from 30% to 60%, with an overall survival rate of approximately 9 months [13].

Compared to de novo AML, the management of s‐AML is more challenging due to inferior clinical outcomes, reduced hematopoietic stem cell reserve, and lower overall survival rates [14].

Herein, we present the case of a 77‐year‐old male who developed secondary acute myelogenous leukemia following RT for rectal cancer. Such cases remain exceptionally rare, with only limited reports in the literature.

## 2 Case

A 77‐year‐old male, a non‐alcoholic smoker with a 15‐pack‐year history, was diagnosed with rectal cancer in 2017.

In February 2023, 5 years post‐diagnosis with rectal cancer, he came to Al‐Biruni University Hospital (Damascus, Syria) with symptoms that began 3 months before admission, including fatigue, general weakness, and progressively worsening exertional dyspnea. He also reported dizziness, headache, blurred vision, loss of appetite, and a weight loss of approximately 10 kg over 6 weeks.

**FIGURE 1:**
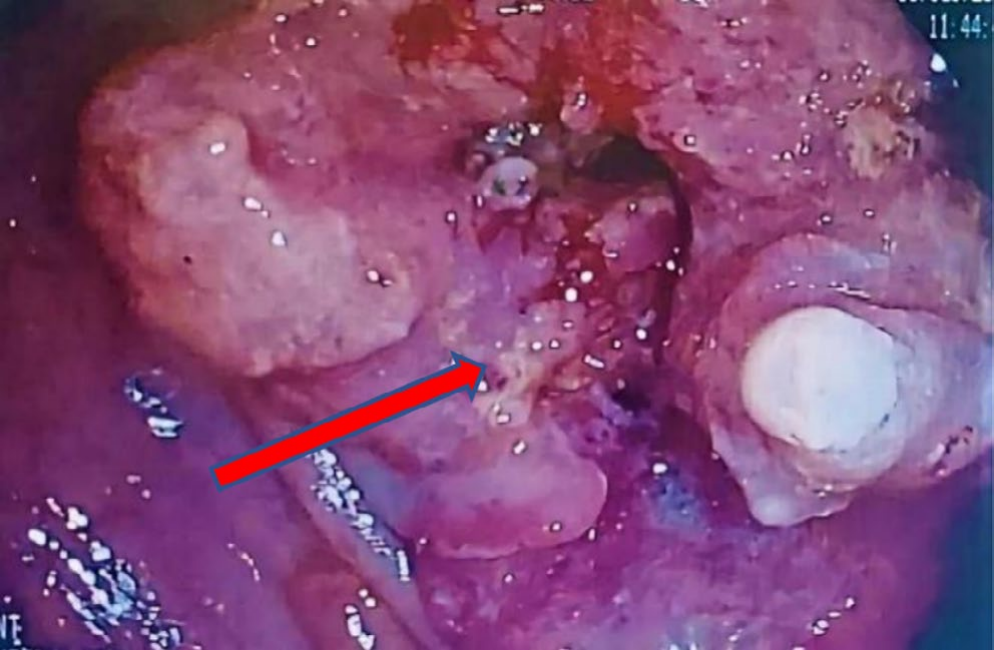
Endoscopic view Colonoscopy image showing a large ulcerated, circumferential rectal mass with infiltrative borders, extensive necrosis, and marked luminal narrowing (arrow). The obstructive morphology is consistent with moderately differentiated rectal adenocarcinoma at diagnosis.

Reviewing the patient's prior history, he had been experiencing alternating bouts of diarrhea and severe constipation. A lower gastrointestinal endoscopy revealed rectal neoplasia, and he subsequently underwent emergency surgery. The sigmoid colon and the upper third of the rectum were resected, along with the removal of regional lymph nodes, ensuring a safety margin of approximately 4 cm from the tumor (Figure 1).

Histopathological examination confirmed a moderately differentiated infiltrating adenocarcinoma that had invaded the muscularis propria and extended into the pericolic fat. There was evidence of metastatic malignancy in the 19 lymph nodes sampled (0/19). Based on these findings, the patient was classified as Colorectal Carcinoma Stage IIA [T3 N0 M0], which denotes a tumor that has penetrated through the muscularis propria into the outermost layers of the colon or rectum (T3), without involvement of regional lymph nodes (N0) and no evidence of distant metastasis (M0). This staging reflects a locally advanced but non‐metastatic disease.

Adjuvant chemoradiation was chosen based on the physician's discretion despite the tumor's node‐negative status due to the tumor's locally advanced characteristics (T3 with pericolic fat invasion) and the potential for locoregional recurrence.

The patient then underwent RT, receiving 45 Gray (Gy) in 25 daily fractions of 1.8 Gy each, delivered over 5 weeks. Following radiation, he received a 6‐month course of 8 cycles of adjuvant CAPOX chemotherapy, consisting of capecitabine (Xeloda) taken orally at 1500 mg twice daily for 14 days, followed by a 7‐day rest, with oxaliplatin given intravenously on Day 1 of each 3‐week cycle.

The patient was placed on regular follow‐up, with an annual endoscopy and CT scans every 6 months.

Five years later, the patient was readmitted with worsening dyspnea on exertion, fatigue, and generalized weakness that had developed over the past 3 months.

Over a month and a half, he also developed blurred vision, headaches, dizziness, and a weight loss of approximately 10 kg.

Additionally, he experienced melena, and his hemoglobin dropped to 4.5 g/dL. A blood transfusion of three units of blood was administered.

**FIGURE 2:**
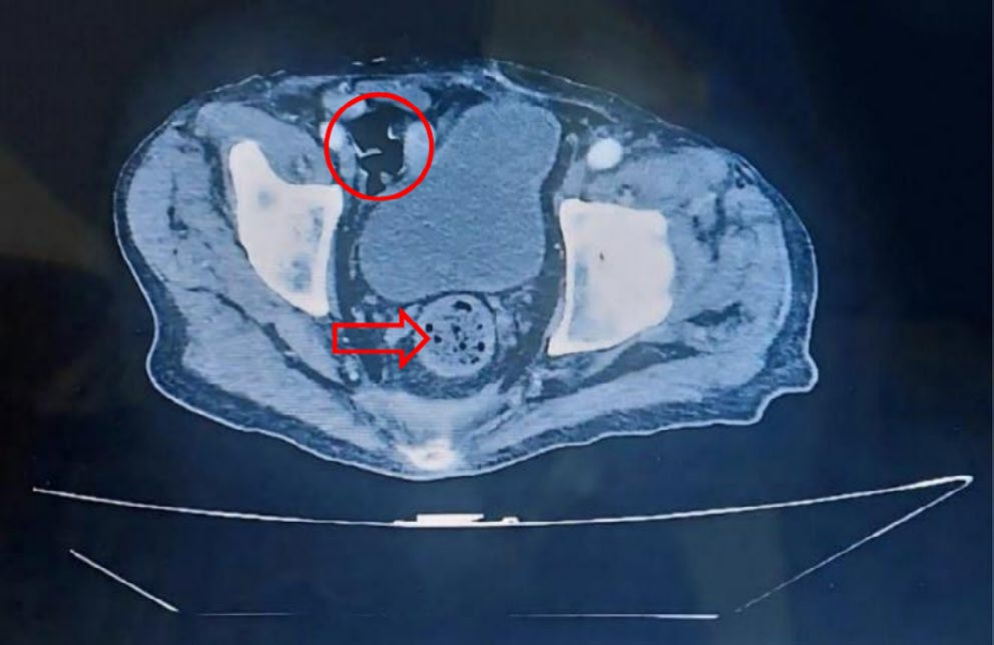
Abdominal CT scan Contrast‐enhanced abdominal CT scan revealing focal asymmetric thickening of the cecal wall (circle), raising suspicion for secondary pathological involvement. Degenerative changes of the first lumbar vertebra are also evident (arrow).

On clinical examination, the patient was noted to have severe pallor, crusts, purpura, and pustules on his skin, but no palpable nodular enlargements were detected at the bases of the lungs. A CT scan of the abdomen and pelvis revealed thickening of the tissue in the cecum and ascending colon, with slight collapse of the first lumbar vertebra and variable degenerative changes in other vertebrae (Figure 2).

**FIGURE 3:**
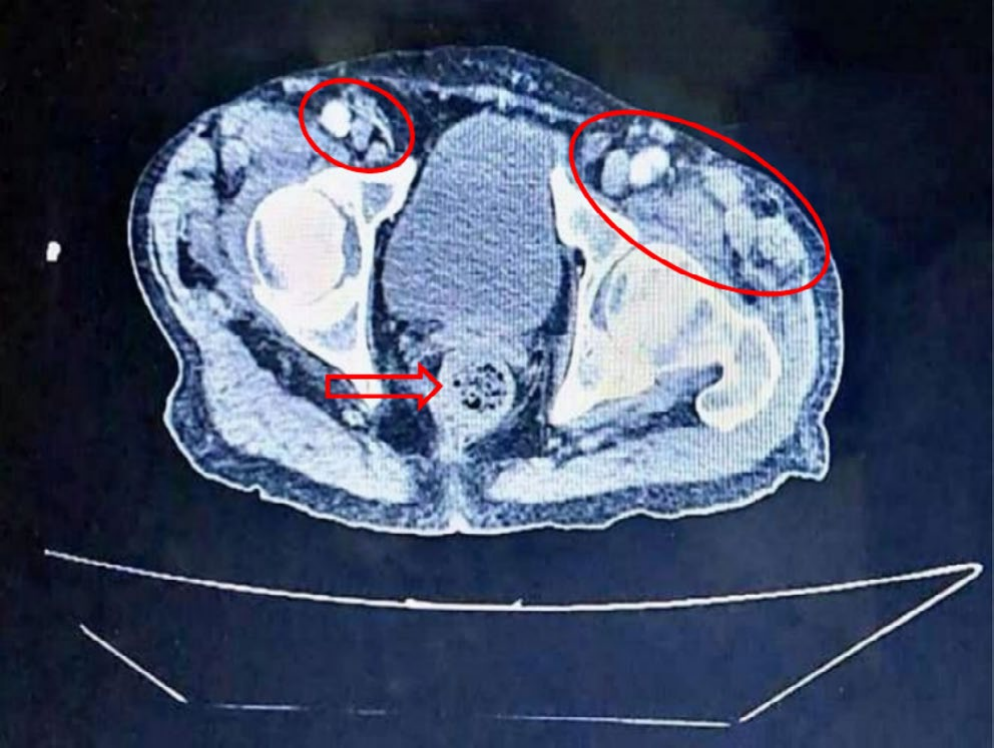
Pelvic CT scan Contrast‐enhanced pelvic CT scan of the pelvis demonstrating multiple enlarged pelvic lymph nodes (circle), the largest measuring 13 × 14 mm. Mild degenerative changes in the lumbar spine are also seen (arrows). Both findings are suggestive of reactive or metastatic involvement.

Multiple nodular hyperplasia were noted in the region, with the largest lymph node measuring 13 × 14 mm (Figure 3); however, none of these lesions raised concern. Biopsies from the ileum and rectum revealed numerous inflammatory ulcers, and antacids and antibiotics were prescribed.

A peripheral blood smear and complete blood count (CBC) showed significant thrombocytopenia and mild anemia (Table 1).

**TABLE 1:** Laboratory results at AML diagnosis.

Category	Test	Result	Normal range
Hematology	Hemoglobin (g/dL)	9.3	13.5–17.5 (M)/12.0–15.5 (F)
	Hematocrit (%)	29	38–50 (M)/34–45 (F)
	White blood cells (×10^9^/L)	5.8	4.0–11.0
	Platelets (×10^9^/L)	12	150–450
	Reticulocytes (%)	1.7	0.5–1.5
	Mean corpuscular volume (fL)	89	80–100
	ESR (mm/h)	80	< 20 (M)/< 30 (F, elderly)
	Differential count (%)	Lymphocytes 37/Neutrophils 8	Lymphocytes: 20–40/Neutrophils: 40–70
Coagulation	PT (s)	79	11–15
	PTT (s)	28	25–35
	INR	1.1	0.8–1.2
Renal function	Urea (mg/dL)	29	7–20
	Creatinine (mg/dL)	1.2	0.7–1.3 (M)/0.6–1.1 (F)
Liver function	AST (U/L)	22	10–40
	ALT (U/L)	6	7–56
	ALP (U/L)	56	44–147
	Albumin (g/dL)	3.2	3.5–5.0
	Total protein (g/dL)	7	6.0–8.3
Iron studies	Iron (μg/dL)	207	60–170
	Ferritin (ng/mL)	9037	30–400 (M)/15–150 (F)
	TIBC (μg/dL)	223	240–450
Vitamins	Vitamin B9 (Folate, ng/mL)	338	> 3
	Vitamin B12 (pg/mL)	> 2000	200–900
Metabolic panel	Sodium (mmol/L)	135	135–145
	Potassium (mmol/L)	4.6	3.5–5.0
	Glucose (mg/dL)	100	70–100 (fasting)
	Cholesterol (mg/dL)	116	< 200
	Triglycerides (mg/dL)	135	< 150
	LDH (U/L)	207	140–280

*Note:* Reference ranges are based on national laboratory standards for adult males.

Abbreviations: ALP, alkaline phosphatase; ALT, alanine aminotransferase; AST, aspartate aminotransferase; ESR, erythrocyte sedimentation rate; INR, international normalized ratio; LDH, lactate dehydrogenase; PT, prothrombin time; PTT, partial thromboplastin time; TIBC, total iron‐binding capacity.

The blood smear demonstrated a marked increase in white blood cells, with 52% comprising immature and abnormal blood cells.

A bone marrow biopsy revealed lymphoid cell infiltration, pan‐hypocellularity of 10%, with no evidence of metastatic disease, consistent with a diagnosis of lymphoproliferative disorder. Immunofluorescent stains (CD117, CD3, TDT, MPO, and CD20) were performed, and the pathology report showed that the peripheral blood sample was composed of over 60% medium‐ to large‐sized blasts with blue cytoplasm, some containing primary cytoplasmic granules. A small number of polymorphonuclear neutrophils were also identified. A population of small mature and some reactive lymphocytes is detected (20%). Monocytes compose 8%–10% total WBCs (noting that co‐expression of CD14/CD117 is negative).

An immunophenotyping panel with microscopic observation can detect immature myeloblasts with a positive expression of CD34 and HLA‐DR and a dim positive expression of CD117. CD13 and CD33 are weakly positive (Table 2).

**TABLE 2:** Immunophenotyping results of bone marrow blasts.

Category	Marker(s)	Expression
Stem cell/Immaturity	CD34	57% (positive)
	HLA‐DR	57% (positive)
	CD117	42% (dim positive)
Myeloid lineage	CD13	22% (weak positive)
	CD33	39% (weak positive)
	MPO	20% (positive)
Lymphoid (T‐cell)	CD2	19% (positive)
	CD3	22% (positive)
	CD7	20% (positive)
Lymphoid (B‐cell)	CD19	2% (positive)
	CD79a	2% (positive, intracellular)
	CD10	0% (negative)
Other markers	CD14	10.6% (positive)
	CD15	12% (positive)
	CD45	99% (positive)
Dual co‐expressions	HLA‐DR/CD34	56%
	CD14/CD117	1%
	CD13/CD33	14%

*Note:* The immunophenotyping profile demonstrated blasts with stem cell and myeloid marker positivity (CD34, HLA‐DR, MPO, CD117), along with aberrant expression of lymphoid antigens (CD2, CD3, CD7). Co‐expression patterns (HLA‐DR/CD34, CD14/CD117, CD13/CD33) were noted but did not alter the diagnostic conclusion. Overall, findings were consistent with immature myeloid blasts, supporting the diagnosis of therapy‐related acute myeloid leukemia (t‐AML).

Abbreviations: CD, cluster of differentiation; HLA‐DR, human leukocyte antigen–DR isotype; MPO, myeloperoxidase.

Based on these findings, the patient was diagnosed with t‐AML, likely induced by prior RT. He received azacitidine (Vidaza), a hypomethylating agent often prescribed in patients who are not eligible for intensive induction chemotherapy or stem cell transplantation. Azacitidine, as a lower‐intensity treatment, is usually better tolerated than standard chemotherapy, which was especially relevant for this 77‐year‐old man with prior exposure to chemotherapy and radiation and clinical signs of frailty. The decision to administer three cycles of azacitidine was guided by his advanced age and overall condition.

Despite receiving three cycles of azacitidine, the patient died 7 months after the initial diagnosis due to septic shock, an unanticipated adverse event.

**FIGURE 4:**
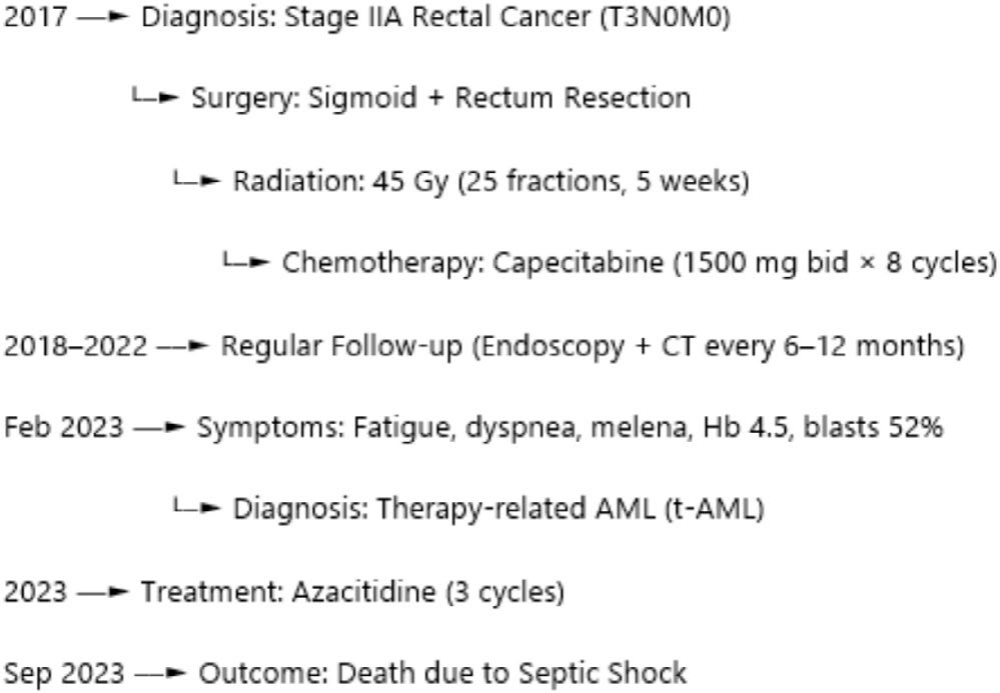
Clinical timeline of the patient's disease course.

A chronological timeline of the patient's disease course is presented in Figure 4.

## 3 Discussion

Alkylating agent‐induced treatment‐related myelodysplastic syndromes (t‐MDS) and acute myeloid leukemia (t‐AML) typically appear 4–7 years after exposure and are frequently associated with genetic abnormalities, particularly those involving chromosomes 5 and 7 [9, 15, 16, 17].

Prognosis is worse than de novo AML, with a 5‐year survival rate of less than 10% of cases due to resistance to chemotherapy, low remission rates, and frequent relapses [18].

Treatment options range from allo‐HSCT to hypomethylating agents, but older patients often cannot tolerate intensive therapy [12].

CPX‐351 has demonstrated superior remission rates and improvements in event‐free and overall survival compared to conventional chemotherapy [19].

In our case, azacitidine was selected over extensive induction chemotherapy or stem cell transplantation due to the patient's advanced age and fragility. The selection of azacitidine is consistent with real‐world clinical practice, which favors hypomethylating agents for older or unfit individuals with secondary AML [20].

During azacitidine treatment, our patient's peripheral blasts showed a transient decline after the first cycle. Still, the patient remained transfusion‐dependent with no remission, and his clinical course was complicated by recurrent infections, culminating in septic shock 7 months later.

Cytotoxic therapies may cause genetic mutations in HSCs, predisposing them to secondary leukemias [21].

Treatments for primary cancers, including radiation and high‐dose alkylating agents or topoisomerase II inhibitors, are known risk factors for secondary malignancies [22, 23, 24].

Our patient developed AML approximately 6 years after treatment for rectal cancer, which included 45 Gy of pelvic radiation and 6 months of CAPOX chemotherapy (oxaliplatin and capecitabine). This represents t‐AML, an uncommon complication affecting less than 10% of individuals receiving cytotoxic therapy [25].

Latency varies about 5–7 years for alkylating agents and 2–3 years for topoisomerase II inhibitors. The role of radiotherapy in AML development is less defined [26].

t‐AML following colorectal cancer is rare, with population‐based studies and institutional cohorts consistently reporting incidences well below 1% among survivors, in contrast to higher rates observed after breast or ovarian cancer treatment [27, 28].

Published reports of t‐AML after solid tumors describe poor outcomes regardless of patient age or the type of initial malignancy [29, 30, 31].

Outcomes are generally poor for cases treated with topoisomerase II inhibitors, such as doxorubicin [32] and etoposide [30, 32], or alkylating agents, like cyclophosphamide and ifosfamide [10, 30, 32]. In most of the reviewed cases, chemotherapy was the primary treatment modality [10, 14, 25, 32, 33], often in combination with RT [25, 29]. However, radiotherapy was not used as a primary treatment in any of these cases, as it is typically reserved for treating metastases [29].

While increased AML risk following early colorectal cancer has been documented in large population‐based studies, only a few individual cases describe t‐AML following adjuvant capecitabine use in colorectal cancer [27, 28]. To our knowledge, t‐AML emerging after combined pelvic radiation and CAPOX chemotherapy for rectal cancer remains exceptionally rare, with virtually no directly comparable cases in the literature.

In this patient, fatal septic shock occurred during azacitidine therapy. Septic complications are an important adverse event in elderly patients receiving hypomethylating agents, underscoring the importance of infection surveillance and supportive care.

The latency period for t‐AML following RT is longer than that for t‐AML caused by chemotherapy alone or in combination with RT [12]. Although RT alone can raise the risk of secondary MDS/AML, the causal relationship may not always be evident due to improvements in modern radiation techniques [34].

It is still unclear how radiation exposure and the development of AML are related to colorectal cancer patients. Further research, including comprehensive karyotypic and histological analyses, is essential to better explain the underlying mechanisms and identify optimal therapeutic strategies for these patients.

This case presents an unusual occurrence of therapy‐related acute myeloid leukemia following combined pelvic radiation and XELOX (capecitabine + oxaliplatin)‐based chemotherapy for colorectal carcinoma. Although therapy‐related AML is well recognized after treatment of hematologic malignancies, its development in the context of colorectal cancer therapy remains rare and insufficiently documented. Therapeutic options were limited due to the patient's advanced age and frailty, along with the development of septic shock during azacitidine therapy. The case highlights the importance of long‐term hematologic surveillance in cancer survivors treated with multimodal therapy and underscores the need for careful infection monitoring during low‐intensity chemotherapy in elderly patients.

## Author Contributions

A.A. contributed to conceptualization and supervision. L.A.H., L.K., and B.S. contributed to methodology, data collection, and manuscript writing and review. S.G.G. contributed to writing, review, and visualization. All authors read and approved the final manuscript.

## Disclosure

The authors confirm that this manuscript has not been published or submitted for publication elsewhere. This submission complies with Cancer Reports policies, which allow prior posting on preprint servers or the authors'’ personal or institutional websites.

## Ethics Statement

Ethical approval was not required for this single‐patient case report according to the policies of the Oncology Department, Damascus University. The report complies with the ethical standards outlined in the Declaration of Helsinki.

## Consent

Written informed consent was obtained from the patient to publish this case report and any accompanying images.

## Conflicts of Interest

The authors declare no conflicts of interest.

## Data Availability

Data sharing is not applicable to this article as no datasets were generated or analysed during the current study.
